# Tau phosphorylation by glycogen synthase kinase 3β modulates enzyme acetylcholinesterase expression

**DOI:** 10.1111/jnc.15189

**Published:** 2020-10-04

**Authors:** María‐Ángeles Cortés‐Gómez, Esther Llorens‐Álvarez, Jordi Alom, Teodoro del Ser, Jesús Avila, Javier Sáez‐Valero, María‐Salud García‐Ayllón

**Affiliations:** ^1^ Hospital General Universitario de Elche FISABIO Unidad de Investigación Elche Spain; ^2^ Centro de Investigación Biomédica en Red sobre Enfermedades Neurodegenerativas (CIBERNED) Madrid Spain; ^3^ Instituto de Neurociencias de Alicante Universidad Miguel Hernández‐CSIC Sant Joan d’Alacant Spain; ^4^ Servicio de Neurología Hospital General Universitario de Elche FISABIO Elche Spain; ^5^ Alzheimer’s Disease Investigation Research Unit CIEN Foundation Queen Sofia Foundation Alzheimer Research Center Madrid Spain; ^6^ Department of Molecular Neuropathology Centro de Biología Molecular 'Severo Ochoa' CBMSO CSIC‐UAM Madrid Spain

**Keywords:** acetylcholinesterase, Alzheimer's disease, cerebrospinal fluid, glycogen synthase kinase‐3β, glycogen synthase kinase‐3β inhibitor, tau phosphorylation

## Abstract

In Alzheimer's disease (AD), the enzyme acetylcholinesterase (AChE) co‐localizes with hyperphosphorylated tau (P‐tau) within neurofibrillary tangles. Having demonstrated that AChE expression is increased in the transgenic mouse model of tau Tg‐VLW, here we examined whether modulating phosphorylated tau levels by over‐expressing wild‐type human tau and glycogen synthase kinase‐3β (GSK3β) influences AChE expression. In SH‐SY5Y neuroblastoma cells expressing higher levels of P‐tau, AChE activity and protein increased by (20% ± 2%) and (440% ± 150%), respectively. Western blots and qPCR assays showed that this increment mostly corresponded to the cholinergic *ACHE‐T* variant, for which the protein and transcript levels increased ~60% and ~23%, respectively. Moreover, in SH‐SY5Y cells differentiated into neurons by exposure to retinoic acid (10 µM), over‐expression of GSK3β and tau provokes an imbalance in cholinergic activity with a decrease in the neurotransmitter acetylcholine in the cell (45 ± 10%). Finally, we obtained cerebrospinal fluid (CSF) from AD patients enrolled on a clinical trial of tideglusib, an irreversible GSK3β inhibitor. In CSF of patients that received a placebo, there was an increase in AChE activity (35 ± 16%) respect to basal levels, probably because of their treatment with AChE inhibitors. However, this increase was not observed in tideglusib‐treated patients. Moreover, CSF levels of P‐tau at the beginning measured by commercially ELISA kits correlated with AChE activity. In conclusion, this study shows that P‐tau can modulate AChE expression and it suggests that AChE may possibly increase in the initial phases of AD.

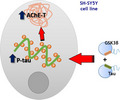

## Graphical abstract

In SH‐SY5Y and mouse primary cells, we have demonstrated that an increased phosphorylation of tau by kinase GSK3β, as occurs in Alzheimer disease, can modulate the levels and enzymatic activity of cholinergic AChE. These changes imply augmented activity, transcript and protein levels of AChE compromising cholinergic transmission. Patients undergoing clinical trial with GSK3β inhibitors showed differential modulation of cerebrospinal fluid‐AChE between placebo and treated patients. Therefore, it is evident the need of analyzing cholinergic enzymes and neurotransmitters in future studies using animal models or clinical trials.

**Abbreviations**
ACh: acetylcholine
AChE: acetylcholinesterase
AChE‐H: acetylcholinesterase “hydrophobic” transcript
AChE‐I: AChE inhibitor
AChE‐R: acetylcholinesterase “readthrough” transcript
AChE‐T: acetylcholinesterase “tailed” transcript
AD: Alzheimer's disease
APPsw: APP Swedish mutation
Aβ: β‐amyloid
ChAT: choline acetyltransferase
CSF: cerebrospinal fluid
FBS: fetal bovine serum
G_1_
: monomers
G_4_
: tetramers
GAPDH: glyceraldehyde 3‐phosphate dehydrogenase
GSK3β: glycogen synthase kinase‐3β
Iso OMPA: tetraisopropyl pyrophosphoramide
N‐AChE‐T and N‐AChE‐R: AChE variants with extended N terminus
NFT: neurofibrillary tangles
P‐tau: hyperphosphorylated tau
QD: once a day
QOD: every other day
RA: all trans‐retinoic acid
T‐tau: total tau

## 1 INTRODUCTION

During the progressive course of Alzheimer's disease (AD), the most common neurodegenerative dementia in the elderly, there is a loss of forebrain cholinergic neurons and a marked synaptic cholinergic deficit that most likely contributes to the cognitive, behavioral, and functional symptoms of AD (Bohnen et al., 2005; Mufson et al., 2003, 2008). This cholinergic deficiency courses with a progressive decline in synaptic acetylcholine (ACh) levels (Davies & Maloney, 1976; Perry et al., 1977), affecting both choline acetyltransferase (ChAT), the rate‐limiting enzyme that synthesizes ACh, and acetylcholinesterase (AChE), the ACh hydrolyzing enzyme, activities. However, AChE activity is increased around the two neuropathological hallmarks of the disease: the amyloid plaques, which are extracellular deposits of aggregated β‐amyloid (Aβ) protein, and the neurofibrillary tangles (NFT) of microtubule‐associated protein tau abnormally hyperphosphorylated (P‐tau) (Mesulam et al., 1987). The increase in AChE in the plaques may be triggered by the deposition of aggregated Aβ, since Aβ peptides influence AChE levels both in vitro (Hu et al., 2003; Sberna et al., 1997) and in vivo (Dumont et al., 2006; Sberna et al., 1998). The early increase in AChE around NTF, even in regions lacking amyloid plaques (Ulrich et al., 1990) has yet to be explained.

AChE is very polymorphic, with different molecular forms adopting distinct subcellular locations and performing a variety of physiological roles (Massoulié, 2002; Meshorer & Soreq, 2006). Alternative mRNA splicing generates three AChE transcripts that differ in their C‐terminal region. The cholinergic AChE form is a tetramer formed by subunits encoded by the AChE‐T (tailed) transcript, which co‐exists in brain with minor amounts of the AChE‐R (“readthrough”) variant whose expression is increased in stress (Meshorer et al., 2002), and AD (Campanari et al., 2016). A third splicing variant AChE‐H (“hydrophobic”), in most species is expressed in non‐nervous cells (Montenegro et al., 2014). Moreover, an alternate upstream promoter usage produces another version of each of these AChE variants with an extended N terminus (N‐AChE‐T and N‐AChE‐R) (Meshorer & Soreq, 2006).

Our group has previously shown that over‐expression of P‐tau leads to an increase in AChE expression in the brain of Tg‐VLW mice expressing 3 missense mutations of human tau (G272V‐P301L‐R406W) associated with the autosomal dominantly inherited frontotemporal dementia and parkinsonism linked to chromosome 17 (FTDP‐17) (Silveyra, et al., 2012). Moreover, AChE activity and mRNA of AChE‐T transcript have been reported to be increased in the septum, but not in other brain areas, of Tg601 mice, a transgenic model of tauopathy that expresses P‐tau‐positive neurons, but does not develop NFTs (Hara et al., 2017). These previous results suggest that an altered tau phosphorylation may underlie some of the changes in AChE levels observed in patients with AD.

The major tau phosphorylating kinase is glycogen synthase kinase‐3β (GSK3β) that may have a central role in the AD pathogenesis (Hooper et al., 2008), as well as in other neurodegenerative diseases (Yang et al., 2008). GSK3β over‐expression and subsequent increased P‐tau, have been correlated with neurodegeneration (Lucas et al., 2001) and formation of NFT (Chu et al., 2017; Jaworski et al., 2011). Moreover, GSK3β inhibitors have been proposed as therapeutic agents for AD (Hooper et al., 2008; Medina & Castro, 2008) since it has been demonstrated that GSK3β inhibition reduces tau phosphorylation (Hooper et al., 2008; Medina & Castro, 2008) and even amyloid production, both in vitro and in vivo (Hooper et al., 2008; Su et al., 2004).

In this study, we have investigated the interaction between AChE and P‐tau in human neuroblastoma SH‐SY5Y cells by analyzing the effect of increased tau phosphorylation mediated by GSK3β on AChE mRNA and protein expression. We also have studied whether inhibition of GSK3β influences AChE expression in neuronal primary cultures. Finally, we have examined AChE activity levels in the cerebrospinal fluid (CSF) of AD patients treated with the GSK3β inhibitor tideglusib (Lovestone et al., 2015; del Ser et al., 2012).

## 2 MATERIALS AND METHODS

### 2.1 Cell culture

SH‐SY5Y human neuroblastoma cells (RRID: CVCL_0019) were cultured in DMEM/F12 + GlutaMAX™‐ I (Dulbecco's Modified Eagle medium, Cat. No. 11,559,726: GIBCO Invitrogen TM Life Technologies) supplemented with 10% heat‐inactivated fetal bovine serum (FBS, Cat. No. 11,580,516: GIBCO Invitrogen TM), penicillin (100 U/mL), and streptomycin (100 μg/mL, Cat. No. 11,528,876: GIBCO Invitrogen TM) and maintained at 37°C in saturated humidity containing 95% air and 5% CO_2_. No sample size calculation was performed, although the number of wells (6 wells for each experimental condition) is in line with those used previously. A maximum of 15 passages were performed. Cells were seeded at a density of 8 × 10 ^5^ cells on 35‐mm tissue culture dishes and were transfected the following day with 4 μg plasmid cDNA per well using Lipofectamine™ 2000 (Cat. No. 11,668,027: InvitrogenTM,) according to the manufacturer's instructions. The plasmid cDNAs employed were as follows: a sequence encoding Xenopus GSK3β inserted into a pcDNA3 vector; the kinase‐dead (K85A) construct of GSK3β (from Jim Woodgett: Addgene plasmid # 14,755; http://n2t.net/addgene:14755; RRID: Addgene_14755); the human wild‐type tau open reading frame encoding the 4‐microtubule‐binding repeat isoform with two N‐terminal inserts, placed in the pSG vector (Montejo de Garcini et al., 1994); the tau triple mutant tau‐VLW also inserted into the pSG vector. The “empty” pCI vector (Cat. No. E1731: Promega) served as a negative control. We also employed a GFP‐PCI vector (Promega) as a control to estimate the transfection efficiency.

After 48 hr from transfection, cells were washed with phosphate‐saline buffer (PBS, Cat. No. D8537: Sigma‐Aldrich Co.) and suspended in 120 µl ice‐cold extraction buffer: 50 mM Tris‐HCl [pH 7.4] (Cat. No. T1503: Sigma‐Aldrich Co.), 150 mM NaCl (Cat. No. S9888: Sigma‐Aldrich Co.), 5 mM EDTA (Cat. No. ED‐500: Sigma‐Aldrich Co.), 1% (w/v) Nonidet P‐40 (Cat. No. I3021: Sigma‐Aldrich Co.), 0.5% (w/v) Triton X‐100 (Cat. No. T9284: Sigma‐Aldrich Co.), supplemented with a cocktail of protease inhibitors (García‐Ayllón et al., 2014). The homogenates were centrifuged at 70,000  *g* at 4°C for 1 hr, and then the supernatants were collected and frozen at −80 ºC until assay. The viability of the transfected cells was assessed in 96‐well plates using the tetrazolium assay (MTS; CellTiter 96® AQueous Assay, Cat. No. G3582: Promega), according to the manufacturer's instructions.

For some experiments, SH‐SY5Y cells were differentiated to cholinergic neurons. Cells were seeded at a density of 3.5 × 10 ^5^ cells on 35‐mm tissue culture dishes and after 24 hr, 10 µM all trans‐retinoic acid (RA, Cat. No. R2625: Sigma‐Aldrich Co.) in DMEM/F12 + GlutaMAX™ with 1% FBS was added. Cells were cultured under these conditions for 8 days, and medium was replaced every 2 days. Then cells were transfected as described earlier.

For confocal assays, Chinese hamster ovary cellular line (CHO cells) that stably over‐expresses AChE‐T (a generous gift of Prof Hermona Soreq) were employed. Cells were cultured in DMEM with stable glutamine (Dulbecco's Modified Eagle Medium, CAPRICORN Scientific, cat. no. DMEM‐HPSTA) supplemented with 10% heat‐inactivated FBS, penicillin (100 U/mL), streptomycin (100 μg/ml) and G418 (50 mg/ml, Sigma‐Aldrich Co., cat. no. G418‐RO), and maintained at 37°C in saturated humidity containing 95% air and 5% CO2. Cells were seeded at a concentration of 50.000 cells/well in a 12‐well sterile culture plate containing one 18mm‐diameter glass coverslip per well and were transfected with GSK3β and tau cDNAs using Lipofectamine™ 2000.

Mouse primary cortical neurons from E16.5 mice embryos were also employed for treatments with GSK3β inhibitors. All procedures and handling of mice were approved by the Ethical Committee of the University Miguel Hernádez (Ref: IN‐MGA‐001–11). Briefly, female pregnant mice (ICR, from inhouse breeding) were killed by decapitation under isofluorane (5% induction, IsoFlo, Ecuphar) anesthesia between 8 and 9 a.m. Cortical lobes from seven mouse embryos were pooled, trypsinized, and dissociated in Hank's balanced salt solution (Cat. No. 14,025,050: Thermo Fisher Scientific). Neurons were plated onto 35‐mm dishes (1.3 × 10^6^ cells/dish) and maintained in Neurobasal medium (Invitrogen, cat. no. 21,103,049) containing B27 supplement (Cat. No. 17,504,044: Gibco BRL), 100 IU/mL penicillin, 100 μg/mL streptomycin, and 2 mM glutamine (Cat. No. 11,500,626: Thermo Fisher Scientific). A total number of three female pregnant mice were used for these experiments. After 7 days in culture, primary cortical neurons were transfected with GSK3β and tau cDNAs using Lipofectamine LTX (Cat. No.15338100: Thermo Scientific), according to the manufacturer's instructions. The efficacy of transfection was assayed using a GFP‐pCI cDNA vector.

### 2.2 Pharmacological treatments

After DNA transfection, SH‐SY5Y cells and primary cortical neurons were treated for 24 hr with 20 μM of SB216763 (Cat. No. S3442: Sigma‐Aldrich Co.), a GSK3β inhibitor (Wagman et al., 2004). After treatment, the conditioned medium was removed and the cells were washed twice with PBS, harvested, suspended in ice‐cold extraction buffer and solubilized as described previously in Cell culture section. Cell viability was assessed using the MTS assay, as described earlier.

### 2.3 AChE assay and total protein determination

AChE activity was assessed with a microassay adapted from the colorimetric Ellman method (Sáez‐Valero et al., 1993), adding 1 mM acetylthiocholine (Cat. No. A5626: Sigma‐Aldrich Co.) in the presence of 50 µM tetraisopropyl pyrophosphoramide (Iso OMPA, Cat. No. T1505: Sigma‐Aldrich Co.) to block any contamination by butyrylcholinesterase. One milliunit (mU) of AChE activity was defined as the number of nmoles of acetylthiocholine hydrolyzed per min at 22°C.

Protein concentrations were determined using the bicinchoninic acid method, with bovine serum albumin as standard (Cat. No. 23,225: Pierce, Rockford, IL).

### 2.4 Sedimentation analysis

Molecular forms of AChE were separated according to their sedimentation coefficients by ultracentrifugation on 5%–20% (w/v) sucrose gradients containing 0.5% (w/v) Triton X‐100 (Sáez‐Valero et al., 1993). Ultracentrifugation was performed in a SW41Ti Beckman rotor at 150,000  *g* for 18 hr at 4 ºC. Approximately 40 fractions were collected from the bottom of each tube and assayed for AChE activity to identify individual AChE forms (G_4_: tetramers; G_1_: monomers) by comparison with the position of molecular weight markers, catalase (11.4S, Cat. No. C9322: Sigma‐Aldrich Co.) and alkaline phosphatase (6.1S, Cat. No. P0114: Sigma‐Aldrich Co.).

### 2.5 Determination of acetylcholine levels

Cellular ACh levels were measured using the commercial fluorometric Choline/Acetylcholine Assay Kit (Cat. No. ab65345: Abcam, Cambridge, UK) following the manufacturer's guidelines. Briefly, SH‐SY5Y cells transfected as previously described were harvested in PBS and re‐suspended in assay buffer. After centrifugation, the levels of total choline and free choline were determined in the supernatant. The concentration of ACh in the samples was calculated as the difference between total choline and free choline levels.

### 2.6 Western blotting assays

The levels of AChE variants, ChAT, tau, P‐tau, GSK3β and were analyzed by immunoblotting. Cellular extracts containing 50 μg protein or 30 µl of human CSF were resolved by electrophoresis on 10% SDS‐polyacrylamide slab gels. Prior to electrophoresis, samples were solubilized in sample buffer and proteins denatured by heating at 98°C for 7 min. Following electrophoresis, proteins were blotted onto nitrocellulose membranes (Cat. No. 41,933: Schleicher & Schuell Bioscience GmbH), the membranes blocked with 5% bovine serum albumin (Cat. No. A3059: Sigma‐Aldrich Co.) and probed with one of the following primary antibodies: mouse anti GSK3β antibody (RRID: AB_10563643: Abcam), mouse anti tau (clone HT7, RRID: AB_2314654: Thermo Fisher), mouse anti P‐tau (Ser202, Thr205; clone AT8, RRID: AB_223647: Thermo Fisher), anti AChE‐T variant (raised against the C terminal amino acid residues 601–614 of human AChE, RRID: AB_722529: Abcam), anti AChE‐R antibody (raised to the unique C‐terminus of human AChE‐R), and anti N‐AChE (raised to the extended N‐terminus of N‐AChE variants). Antibodies against AChE‐R and N‐AChE were a generous gift from Prof Hermona Soreq from The Institute of Life Science, The Hebrew University of Jerusalem, Jerusalem, Israel. A rabbit anti‐glyceraldehyde 3‐phosphate dehydrogenase (GAPDH) antibody (RRID: AB_307275: Abcam) was used as a loading control. Western blots for different antibodies were performed separately avoiding re‐probing of blots. The blots were incubated with the corresponding secondary antibody (Goat anti‐mouse IgG, RRID: AB_2536527; Goat anti‐rabbit IgG, RRID: AB_2536530; Rabbit anti‐Goat IgG, RRID: AB_2556529: Thermo Scientific) conjugated to horseradish peroxidase and the immunoreactive signal was detected using SuperSignal West Dura Extended Duration Substrate (Cat. No. 34,075: Thermo Fisher Scientific) according to the manufacturer's instructions in a Luminescent Image Analyzer LAS‐1000 Plus (FUJIFILM). For semi‐quantitative analysis, protein levels were normalized to GADPH and the intensity of bands was measured by densitometry with the Science Lab Image Gauge v4.0 software provided by FUJIFILM.

### 2.7 RNA isolation and analysis of AChE transcripts by qRT‐PCR

Total RNA from cell cultures was isolated using the DNeasy Blood & Tissue Kit (Cat. No. 69,506: Qiagen) on the QIAcube apparatus (Qiagen) according to the manufacturer's instructions. First‐strand cDNAs were synthesized by reverse transcription of 1.5 µg of total RNA using the High Capacity cDNA Reverse Transcription Kit (Cat. No. 10,186,954: Applied Biosystems; Life Technologies Paisley, UK), according to the manufacturer's instructions. Quantitative reverse transcription‐polymerase chain reaction (*q*RT‐PCR) amplification was performed using StepOne‐Plus™ Real‐Time PCR System with Power SYBR® Green PCR Master Mix (Cat. No. 4,368,577: Applied Biosystems) according to the manufacturer's instructions for analysis of AChE transcripts. The primers used were as follows: AChE‐T forward 5′‐CTTCCTCCCCAAATTGCTC‐3′, reverse 5′‐TCCTGCTTGCTGTAGTGGTC‐3′; AChE‐R forward 5′‐CTTCCTCCCCAAATTGCTC‐3′, reverse: 5′‐GGGGAGAAGAGAGGGGTTAC‐3′; N‐AChE forward 5′‐GAAAGTCCGAAGTCACCCGTC‐3′, reverse 5′‐CAGGCGGCGTCTGAGAA‐3′; GAPDH forward 5′‐AGCCACATCGCTCAGACAC‐3′, reverse 5′‐GCCCAATACGACCAAATCC‐3′. Transcript levels were calculated by the comparative 2^−ΔCt^ method with respect to GAPDH cDNA.

### 2.8 Confocal microscopy

Interaction of P‐tau and AChE was assayed by immunocytochemistry on CHO‐AChE‐T cells. Briefly 48 hr after transfection cells were fixed with methanol and after blocking, they were incubated with a rabbit anti‐AChE N‐terminal antibody (Sigma‐Aldrich, RRID: AB_10669297) and mouse anti P‐Tau (Thr181) monoclonal Antibody (clone AT270; ThermoFisher Scientific, RRID: AB_223651). This antibody recognized tau phosphorylated at Thr181 residue, which is also phosphorylated by GSK3β. Then cells were incubated with secondary antibodies Alexa Fluor® 488‐labeled goat anti‐rabbit IgG (RRID: AB_143165) and Cy5™ goat anti‐mouse IgG (RRID: AB_2534033) and nuclei stained with HOECHST (Sigma‐Aldrich Co., cat. no. D9542). Dual immunofluorescence images were captured with sequential scans using a Leica laser‐scanning spectral vertical confocal microscope (Leica DM2500) and images digitalized and adjusted using Imaris (v9.3, RRID: SCR_007370) software.

### 2.9 Human CSF samples

This study was approved by the ethics committee of the Hospital General Universitario de Elche (License no: PI 10/2011) and was carried out in accordance with the Declaration of Helsinki. This study was not pre‐registered. The CSF samples used for this study were de‐identified leftover aliquots from the ARGO study, a phase II clinical trial with the GSK3β inhibitor tideglusib (NP031112‐010B04) performed by Noscira SA (Madrid, Spain) on AD patients (ClinicalTrials.gov number NCT01350362; see ( ; Lovestone et al., 2015) for details about the study). The total length of the trial was 26 weeks in which AD patients were orally administered 1,000 mg of tideglusib using two different regimes, either once a day (QD; seven cases) or every other day (QOD; seven cases) or a matching placebo (five cases). See Table 1 for details.

**Table 1:** Demografic data and I‐AChE treatment

Treatment	Patient	Tideglusib administration	Age (y)	Gender	AChE‐I treatment	AChE‐I treatment duration (y)
Placebo	1		72	M	Donepezil	2.0
	2		80	M	Donepezil	5.0
	3		83	M	Rivastigmine	0.8
	4		61	F	Donepezil	2.0
	5		78	F	Galantamine	2.0
Tideglusib	6	QOD	64	M	Galantamine	1.1
	7	QOD	59	F	Rivastigmine	1.0
	8	QD	68	F	Galantamine	3.2
	9	QOD	78	M	Rivastigmine	0.4
	10	QD	66	M	Donepezil	3.4
	11	QOD	71	M	Rivastigmine	1.3
	12	QOD	63	M	Rivastigmine	0.5
	13	QD	81	M	Rivastigmine	1.9
	14	QD	73	M	Galantamine	0.9
	15	QD	68	M	Rivastigmine	1.1
	16	QOD	76	M	Donepezil	1.8
	17	QD	68	F	Donepezil	2.4
	18	QD	65	F	Donepezil	4.81
	19	QOD	77	M	Donepezil	1.93

AD patients were administered with placebo or 1,000 mg of tideglusib orally using two different regimes: once a day (QD) and every other day (QOD).

F/M, female/male. All AD patients were under AChE inhibitor (AChE‐I) treatment for at least 4 months prior to the enrollment into the trial. AChE‐I tratmen duration (years, Y) is indicated.

All AD patients (6 females/13 males; 71 ± 2 years old) fulfilled the NINCDS‐ADRDA criteria for “probable” AD (McKhann et al., 1984), had a mean MMSE score of 19 ± 1, had been taking a stable and well‐tolerated dose of an AChE inhibitor (AChE‐I: rivastigmine, donepezil, or galantamine; see Table 1) for at least 4 months prior to the enrollment into the trial, and AChE‐I treatment was continued during the trial.

CSF was obtained by lumbar puncture at baseline and week 26, it was centrifuged at 1,000 *g* for 15 min to eliminate cells and insoluble material and immediately stored at −80ºC. Assays were performed blind for the treatment assignment and no randomization was performed to allocate subjects in the study. The study was exploratory, and no exclusion criteria were pre‐determined with the samples obtained from Noscira.

### 2.10 Determination of core AD biomarkers

The levels of the core AD biomarkers: Aβ_1–42_, total tau and phosphorylated P181 tau, were determined in CSF aliquots using commercial enzyme‐linked immunosorbent ELISA assays (Aβ_1–42_, Cat. No. 81,583; total tau, Cat. No. 81,579; and P181 tau, Cat. No. 81,581: Innogenetics/Fujirebio) following the manufacturer's instructions.

### 2.11 Statistical analysis

All data were analyzed using SigmaStat (Version 3.5, RRID: SCR_010285: Systac Software Inc.). Results passed normality test using the Kolmogorov–Smirnov test. Comparisons between two groups were performed with Student's unpaired *t*‐test or, for multiple comparisons, by one‐way ANOVA followed by a Turkey's post hoc test. Results are presented as means ± *SEM*, with *p* <.05 considered to be significant. No sample calculation was performed.

## 3 RESULTS

### 3.1 Increased tau phosphorylation lead to increased AChE levels

**Figure 1:**
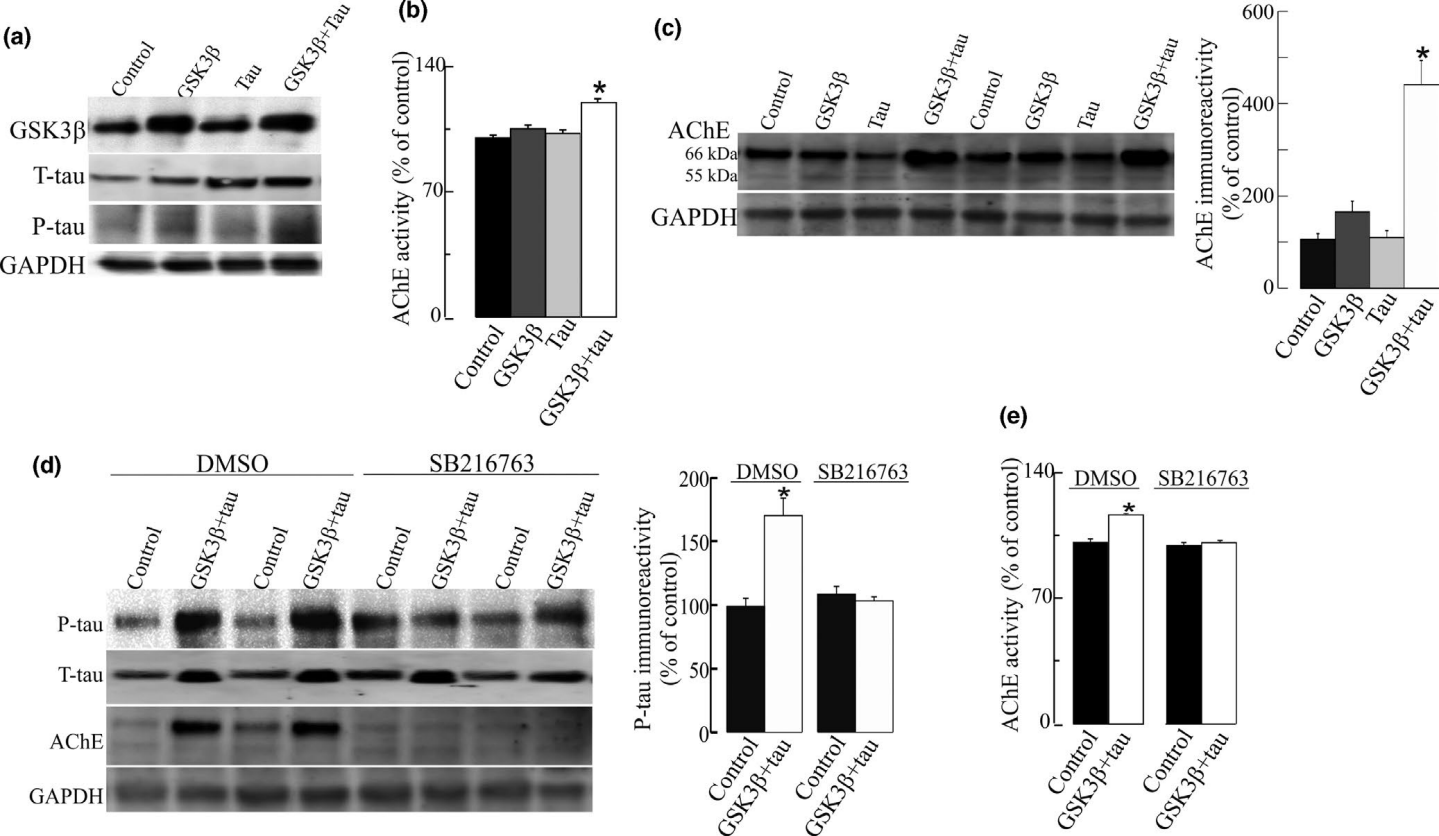
Increase in acetylcholinesterase (AChE) levels in neuroblastoma SH‐SY5Y cells with increased tau phosphorylation as a result of GSK3β and tau over‐expression. SH‐SY5Y cells were transfected with DNA vectors that encode GSK3β, wild‐type tau (tau), or both proteins (GSK3β + tau) or with a pCI empty control vector. (a) Each lane contained 30 µg of protein from cell extracts. Proteins were resolved by electrophoresis and probed with specific primary antibodies to GSK3β, tau or P‐tau (clone AT8). Representative blots are shown. Increased tau phosphorylation was observed in cells that over‐express GSK3β + tau. (b) AChE‐specific activity (mU/mg of total protein) was also determined in cellular extracts. Percentages (%) of AChE activity relative to pCI control cells are represented. (c) Fifty microgram protein extracts were then assayed by immunoblotting using the N‐19 antibody that recognizes all the AChE variants. Representative western blot (left panel) and densitometric quantification (right panel) expressed as percentage (%) relative to immunoreactivity of the control group are shown. Immunoreactivity values obtained by densitometry were normalized relative to that of the housekeeping protein GAPDH. (d) SH‐SY5Y cells transfected with a pCI empty vector or GSK3β + tau were treated with 20 μM of SB216763, a GSK3β inhibitor, or with vehicle (DMSO) for 24 hr. Inhibition of tau phosphorylation was quantified by a decrease in P‐tau immunoreactivity. AChE protein expression was also analyzed by western blots probed with N‐19 antibody. Representative blots are shown. (e) AChE enzymatic activity was assayed in cells treated with 20 μM of SB216763, and expressed as percentage (%) relative to control cells. Results were confirmed in *n* = 18 independent cell determinations (obtained from 3 independent cell sets of experiments). Represented values are means ± *SEM*. *Significantly different (*p* <.05) from the control group, as assessed by one‐way ANOVA followed by Tukey test for pair‐wise comparisons

SH‐SY5Y cells express AChE (Silveyra, et al., 2012; Silveyra, et al., 2012) and have been used to study the effects of tau and GSK3β over‐expression (Bijur et al., 2000; Katsinelos et al., 2018). Thus, in this study, human wild‐type tau and GSK3β were over‐expressed in SH‐SY5Y cells to increase cellular levels of P‐tau as compared with cells transfected with a control pCI “empty” vector or only with either tau or GSK3β alone (Figure 1a). Viability assays showed there was no significant cell death of GSK3β + tau over‐expressing cells (11 ± 5% reduction relative to pCI, *p* =.1).

When AChE enzymatic activity was determined later in the cellular extracts, the levels of AChE were increased (20 ± 2%; *p* <.001) in cells over‐expressing both tau and GSK3β compared with cells over‐expressing tau or GSK3β alone (Figure 1b). AChE protein levels were also determined by western blot using the N‐19 antibody, raised against a peptide that recognizes the N‐terminus of human AChE, which is common to all variants. A major immunoreactive band of ~66 kDa, consistent with the molecular mass of full‐length AChE, and a faint band of 55 kDa that could not be quantified reproducibly, were detected in all samples. Immunoreactivity of the 66 kDa was elevated (440 ± 150% increase, *p* <.001) in cells over‐expressing GSK3β and tau (Figure 1c) compared with pCI control cells. Moreover, a non‐significant increase in AChE immunoreactivity (165 ± 34; *p* = .075; Figure 1c) was also observed in GSK3β over‐expressing cells. To confirm the influence of tau phosphorylation on AChE, SH‐SY5Y cells over‐expressing both GSK3β and tau were treated for 24 hr with 20 μM of the specific GSK3β inhibitor SB216763 (Wagman et al., 2004). The inhibition of tau phosphorylation prevented the changes in AChE activity and protein levels (Figure 1d) in SB216763‐treated cells. Moreover, cells were transfected with a kinase‐dead (K85A) construct of GSK3β (mGSK3β) with tau and no increments on P‐tau were observed (Figure S1a). Therefore, no changes in AChE activity and protein were noticed in mGSK3β + tau over‐expressing cells relative to the controls (Figure S1b).

**Figure 2:**
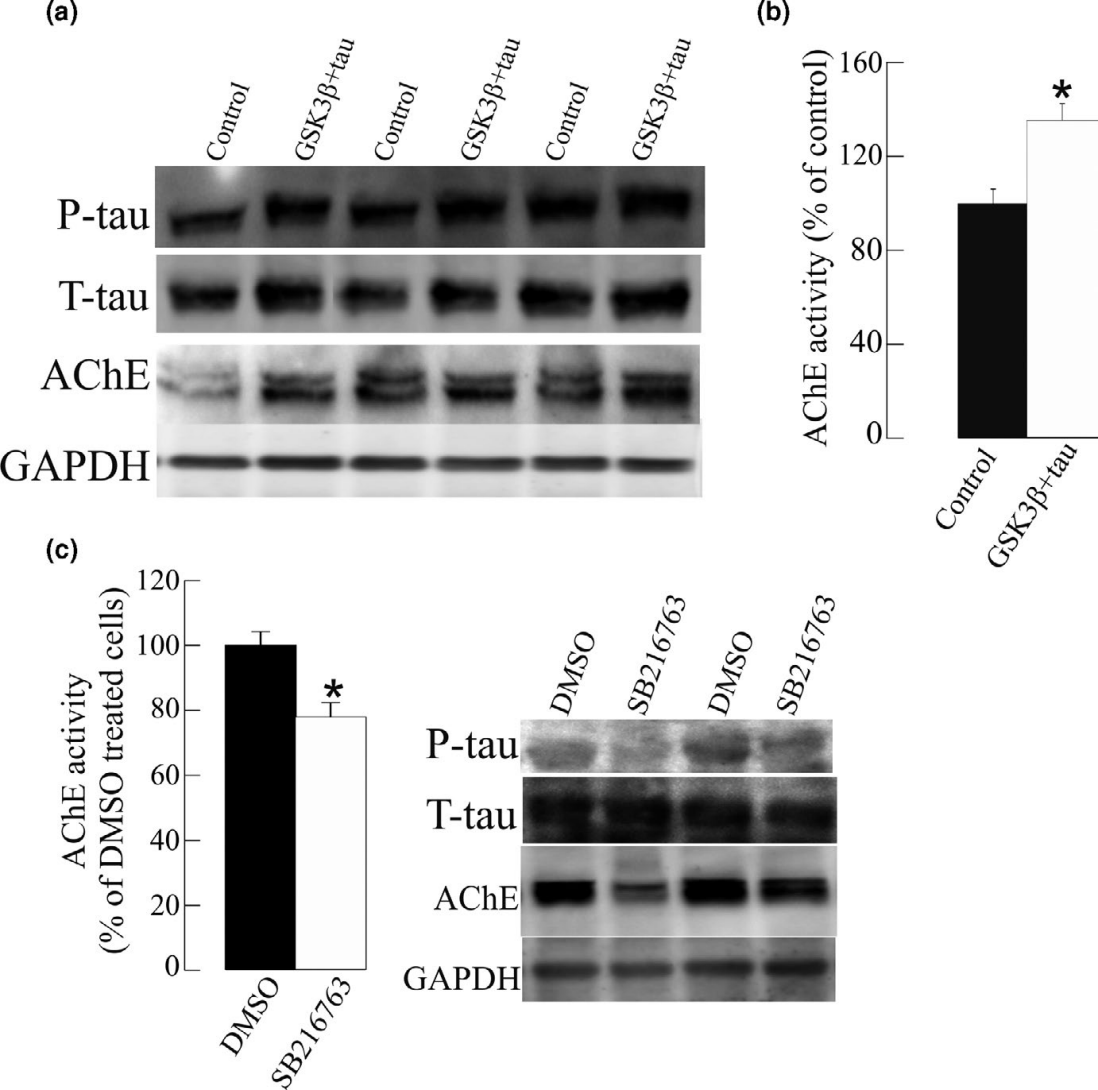
Glycogen synthase kinase‐3β (GSK3β) inhibition by SB216763 decreased acetylcholinesterase (AChE) activity in primary cultured cortical neurons. (a) Over‐expression of GSK3β and tau in primary cortical neurons (GSK3β + tau) resulted in increased P‐tau levels, leading to an increase in AChE protein (blots probed with the N‐19 antibody) and activity, expressed relative to the controls transfected with the empty pCI vector. (b) Neurons transfected with GSK3β + tau were treated for 24 hr with the GSK3β inhibitor SB216763 or the vehicle alone (DMSO; control). A decrease was observed in the AChE protein (blots probed with the N‐19 antibody) and in AChE activity relative to the controls. Mean values ± *SEM* are represented of at least of 18 independent measurements from three independent experiments: *Significantly different from the control group (*p* <.05), as assessed by the Student's *t* test

The effect of P‐tau over‐expression on AChE was also tested in primary cultures of mice neurons. The efficacy of transfection was 10%–15%. Increment in P‐tau as a result of GSK3β and tau over‐expression caused a significant increase in AChE activity and protein levels (Figure 2). The effect of the inhibition of GSK3β by SB216763 on AChE was further confirmed by a significant reduction (23 ± 4% decrease, *p* = .002) of AChE enzymatic activity in cellular extracts treated for 24 hr with SB216763. This result parallels the previous findings of decreased AChE immunoreactivity (Figure 1).

Finally, we also studied the effect of the tau‐VLW mutant on AChE expression. Tau‐VLW carries three mutations that have been identified in patients with frontotemporal dementia and parkinsonism linked to chromosome 17 (FTDP‐17): G272V, P301L, and R406W. Over‐expression of VLW increased P‐tau levels (Figure S2a) and AChE activity (a 14% ± 3 increment, *p* < 0 .001: Figure S2b), as compared with pCI‐transfected cells. Moreover, the AChE protein also increased in these cells as a result of VLW transfection (385% ±43, *p* < .001: Figure S2c).

### 3.2 Cholinergic AChE‐T is the splicing variant increased in cells over‐expressing GSK3β and tau

**Figure 3:**
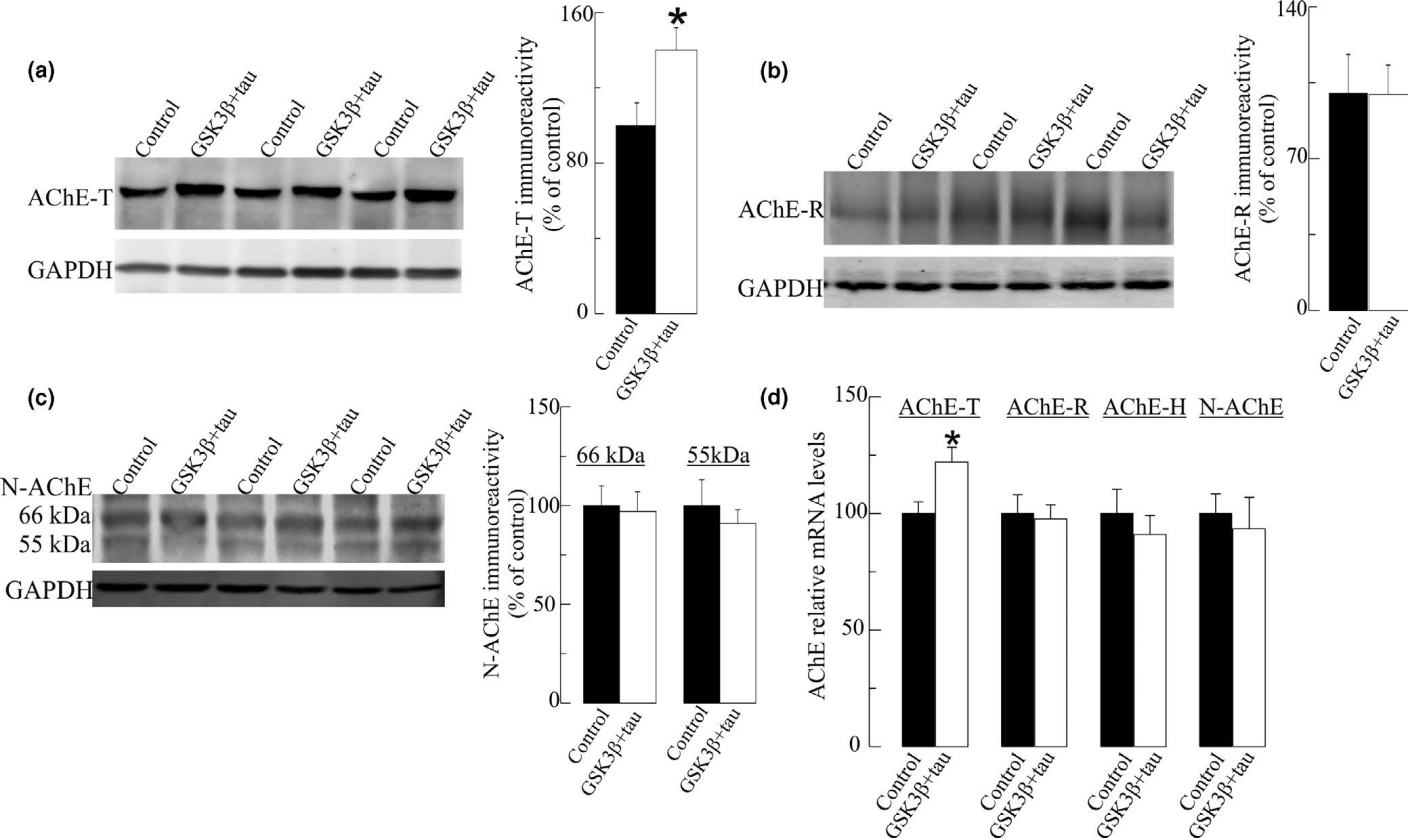
Increased levels of "tailed" acetylcholinesterase (AChE‐T) splice variant in SH‐SY5Y cells with increased tau phosphorylation. Immunodetection of AChE variants in cellular extracts from SH‐SY5Y cells with elevated P‐tau resulting of glycogen synthase kinase‐3β (GSK3β) plus total tau transfection (GSK3β + tau) and control cells transfected with a pCI empty vector (control). Thirty microgram of protein from cell extracts was resolved by electrophoresis and probed with specific primary antibodies raised to: (a) the C‐terminus of the AChE‐T variant; (b) the C‐terminus of “readthrough” acetylcholinesterase (AChE‐R) variant; (c) the extended N‐terminus of AChE (N‐AChE) variants. Representative blots and densitometric quantification of the immunoreactive bands, expressed as percentage (%) relative to immunoreactivity of the control group are shown. For semiquantitative analysis, the levels were normalized to the housekeeping protein GAPDH. (d) Relative mRNA levels of the transcripts for AChE splice variants were analyzed by *q*RT‐PCR. The specificity of the PCR products was confirmed by dissociation curve analysis. Transcript levels were calculated by the comparative 2^−ΔCt^ method with respect to GAPDH. Values are means ± *SEM* from at least of 24 cell wells independent determinations from four independent cell culture experiments. *p <.05 significantly different from the control group (Student's *t*‐test)

The influence of P‐tau on particular AChE variant/s was further analyzed in SH‐SY5Y cells transfected to have higher levels of P‐tau. To study the expression pattern of AChE variants, the levels of each AChE variant were firstly analyzed by SDS PAGE/western blotting using specific anti‐AChE antibodies raised against peptides that recognize the C‐terminus of AChE‐T or AChE‐R. The AChE‐T species were detected as a ~66 kDa immunoreactive band (Figure 3a) which showed higher immunoreactive levels (60 ± 10% increase, *p* < .001) in cells that over‐express GSK3β and tau, compared with cells that over‐express the pCI control vector. The levels of the immunoreactive AChE‐R subunit, resolved as a ~55 kDa band, were not significantly different between control and P‐tau over‐expressing cells (Figure 3b). To our knowledge, no antibodies specific for AChE‐H have been developed. The levels of the N‐extended species were also analyzed with an antibody raised against the extended N‐terminal domain of AChE, which is common to all N‐AChE subunits. This antibody cannot distinguish between N‐extended variants and resolved a predominant band of ~66 kDa and a faint band of ~55 kDa, (Figure 3c). No differences in immunoreactivity for both N‐extended species of AChE were detected between cells that over‐express P‐tau compared with control cells.

*q*RT‐PCR assays were further performed to determine whether alterations in AChE levels corresponded to changes in specific AChE transcripts (Figure 3d). Consistent with western blot analysis, the levels of the cholinergic AChE‐T transcript were higher (23 ± 6% increment, *p* =.01) in cells transfected with GSK3β and tau. In this regard, no differences were detected in the levels of the transcripts encoding the AChE‐R variant nor those that encode all N‐AChE extended variants between cells which over‐express both GS3Kβ and tau and control cells. The levels of the non‐cholinergic AChE‐H transcript, also expressed in SH‐SHY5Y cells (Bi et al., 2014), were also measured and no changes were found in GSK3Β and tau over‐expressing cells compared with controls (Figure 3d). In summary, these results suggested that a specific increase in the expression of the AChE‐T cholinergic species can be triggered by increased phosphorylation of tau by GSK3β.

Undifferentiated SH‐SHY5Y cells express monomeric forms of AChE‐T, and only after differentiation to a neuronal phenotype SH‐SHY5Y cells express a pattern AChE isoforms similar to that in cholinergic neurons, with monomers (G_1_) and tetramers (G_4_) of the AChE‐T variants (Grisaru et al., 1999; Massoulié, 2002; Taylor & Radic, 1994). Thus, AChE activity and protein levels in RA‐differentiated SH‐SY5Y cells were analyzed to further investigate whether P‐tau influences AChE cholinergic species.

**Figure 4:**
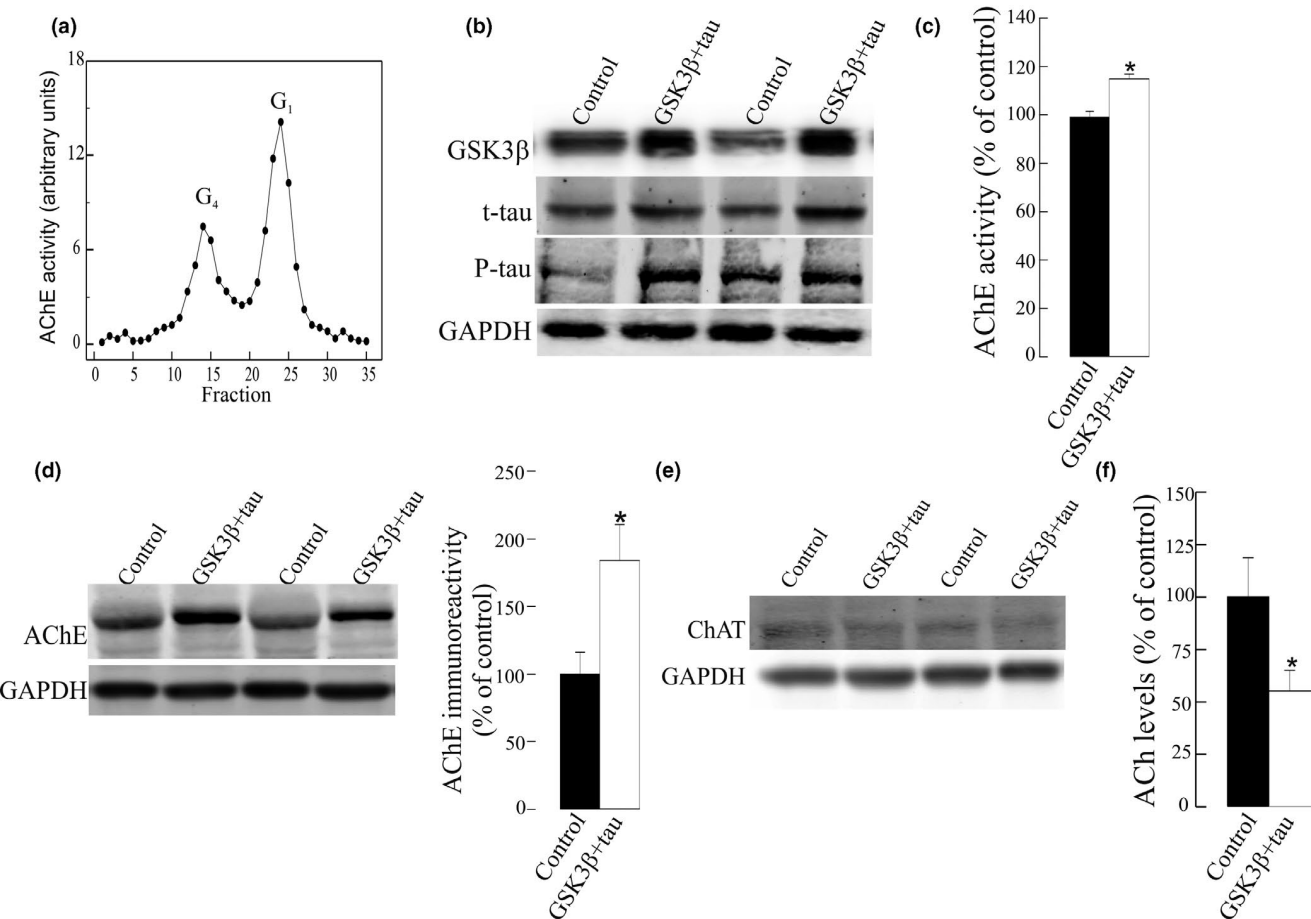
Increased tau phosphorylation leads to a cholinergic imbalance with decreased acetylcholine levels in retinoic acid (RA)‐differentiated SH‐SY5Y cells. RA‐differentiated SH‐SY5Y cells were transfected with pCI empty vector (control) or plasmid cDNAs that encode GSK3β and tau (GSK3β + tau). (a) Ultracentrifugation on sucrose gradient served to verify the expression of tetramers of acetylcholinesterase (AChE) in non‐transfected RA‐differentiated cells. Molecular forms of AChE (tetramers: G_4_; and light monomers: G_1_) were identified by comparison with the position of molecular weight markers catalase (11.4S) and alkaline phosphatase (6.1S). Representative profile is shown. (b) Immunoblots probed to total tau (T‐tau), GSK3β and phosphorylated tau (P‐tau) were then performed to verify transfection and the effect on tau phosphorylation. Equal amounts of protein were loaded in all lanes. Representative blots are shown. (c) AChE enzymatic activity was assayed in cellular extracts and expressed as percentage (%) of the activity respect to pCI control‐transfected cells. (d) AChE protein levels were assayed by western blotting of 30 μg of protein from cellular extracts using the anti‐AChE antibody N‐19. Immunoreactivity of the AChE bands was quantified and normalized to glyceraldehyde 3‐phosphate dehydrogenase (GAPDH). Immunoreactive levels were expressed as % of control levels. Representative blot and densitometric quantification are presented. (e) The levels of choline acetyltransferase (ChAT) were assayed by western blot in cellular extracts, with GAPDH as loading control. (f) Cellular levels of the neurotransmitter acetylcholine (ACh) were also measured using a commercial fluorometric method and expressed as % respect to control. Results were confirmed in at least of *n* = 18 cell wells from three independent cell cultures experiments. Mean value ± *SEM* are represented. *Significantly different (*p* < .05) from the control group, as assessed by the Student's *t* test

First, we confirmed by sedimentation analysis that RA‐differentiated SH‐SY5Y cells express both tetrameric and monomeric AChE (Figure 4a). Then, RA‐differentiated SH‐SY5Y cells were transfected with tau and GSK3β cDNAs to increase cellular levels of P‐tau (Figure 4b). P‐tau levels increased after GSK3β + tau transfection in RA‐differentiated SH‐SY5Y cells, albeit to a lesser extent than in undifferentiated cells, probably because of a lower transfection efficacy in differentiated cells (15%–20%). Like the undifferentiated cells, over‐expression of GSK3β and tau led to an increase in both AChE activity (15 ± 2% increment, *p* < .001: Figure 4c) and AChE protein (184 ± 26% increase, *p* = .04; Figure 3d), as compared to control cells. Sedimentation analysis showed that the ratio of G_4_/G_1_ species which indicates the proportion of tetrameric (G_4_) molecules versus monomeric (G_1_) AChE forms were similar between cells transfected with tau and GSK3β and those with pCI‐control vector (profile not shown). Hence, the increase in AChE in RA‐differentiated SH‐SY5Y cells over‐expressing P‐tau involved an increase in the amount of both molecular forms, monomers and cholinergic tetramers.

We also investigated whether other cholinergic enzymes are influenced by tau phosphorylation. The levels of the acetylcholine‐synthesis enzyme ChAT were assessed by western blotting. However, no differences were found between RA‐differentiated SH‐SY5Y cells over‐expressing GSK3β + tau and control cells (Figure 4e). We further studied whether the increase in AChE might result in an alteration in the levels of the neurotransmitter ACh. Cellular ACh levels were measured using a fluorometric method (Figure 4f). A reduction in ACh levels (45 ± 10% decrease; *p* = .04) was observed in RA‐differentiated SH‐SY5Y cells that over‐express GSK3β + tau compared to control cells.

### 3.3 AChE co‐localizes with P‐tau in cytoplasmatic regions

**Figure 5:**
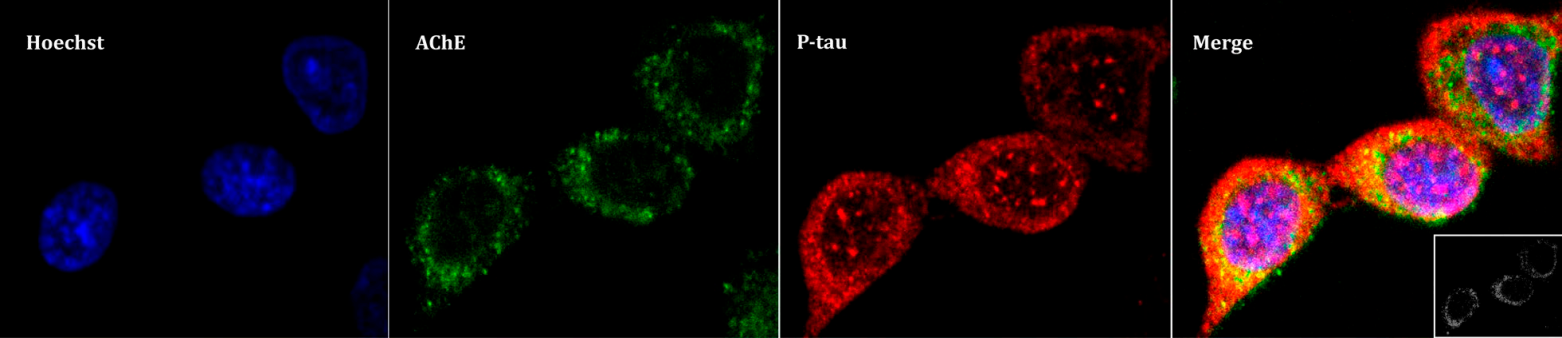
Acetylcholinesterase (AChE) colocalizes with phosphorylated tau (P‐tau) in cytoplasmatic regions in Chinese hamster ovary (CHO) cells. CHO cells that stably over‐express ACHE‐T variant were transfected with plasmid cDNAs that encode glycogen synthase kinase‐3β and tau to increase P‐tau levels. Immunoassay was performed and confocal images were obtained with a x63 oil immersive objective lens. From left to right we observe: stained nuclei with Hoechst in blue; AChE probed with anti‐N‐terminus AChE antibody plus anti‐rabbit IgG Alexa Fluor 488 in green; P‐tau with anti‐PHF‐tau antibody combined with an anti‐mouse IgG Cy5 in red; Merge image showing three channels overlay with a colocalization of P‐tau and AChE image insert on the left‐bottom showing pixels positive for both P‐tau and AChE marked in white. Representative images of *n* = 3 experiments are showed (scale bar = 5 µm)

We have also analyzed the cellular location of AChE and P‐tau to compare their distribution by immunocytochemistry on CHO cells that stably over‐expresses AChE‐T, and P‐tau levels were induced by transfection of GSK3β and tau. P‐tau immunochemistry was performed using an anti p‐T181‐tau antibody instead of an anti‐Ptau202/205AT8 antibody. Previous western blot assays probed with anti p‐T181‐tau antibody also showed the increment on P‐tau levels in GSK3β and tau over‐expressing cells (data not shown). Confocal microscopy analysis (Figure 5) showed that P‐tau co‐localizes with AChE mainly in cytoplasmatic regions with a Mander's coefficients of 0.51 ± 0.09 for P‐tau and 0.41 ± 0.09 for AChE co‐localization with P‐tau.

### 3.4 GSK3β inhibitor treatment influences AChE levels in AD patients

The in vitro studies have shown that an increase in P‐tau levels in a cellular model triggers an increment in AChE expression and that treatment with the GSK3β inhibitor SB216763 can block this effect. To assess the role of tau phosphorylation on AChE in AD patients, CSF samples from patients enrolled in a clinical trial of the GSK3Β inhibitor tideglusib (NP031112‐10B04)) were analyzed. CSF levels of core AD biomarkers, Aβ42, T‐tau, and P‐tau, as well AChE activity were measured in aliquots of CSF samples collected before and after 26 weeks of tideglusib treatment (see Table 1 for details). In agreement with previous results (Lovestone et al., 2015), a non‐significant (*p* =.087) decreasing trend was observed in CSF P‐tau levels after treatment with tideglusib, compared with placebo‐treated patients, whereas no changes were found in the levels of T‐tau (*p* =.84) and Aβ42 (*p* =.14: Figure S3). No differences were found for these core AD biomarkers between QOD and QD treatment administration groups.

**Figure 6:**
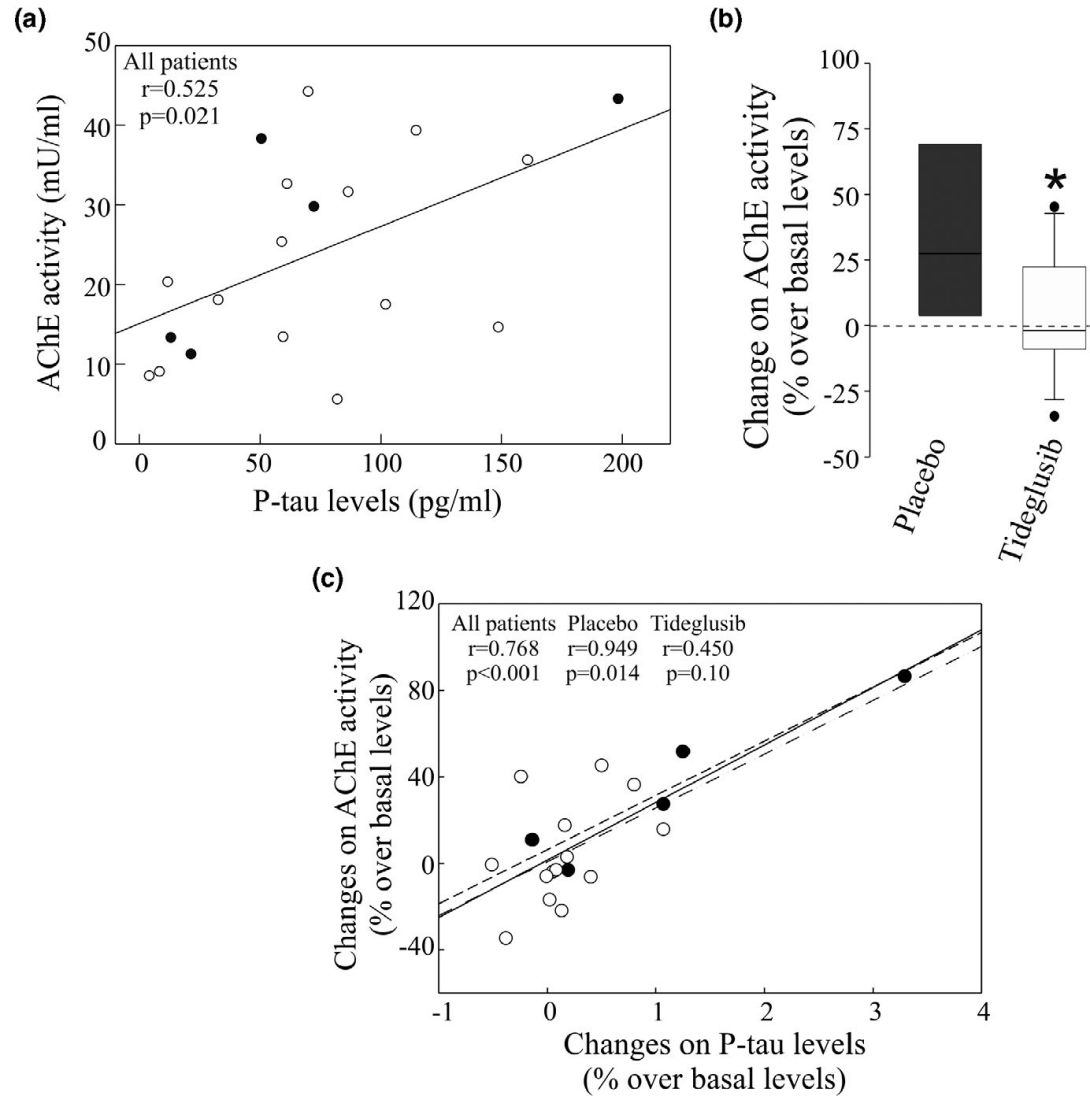
Phosphorylated tau (P‐tau) levels in human CSF correlates with acetylcholinesterase (AChE) enzymatic activity. CSF samples were obtained from Alzheimer's disease (AD) patients treated with tideblusib (*n* = 14; open circles) or with a placebo compound (*n* = 5; closed circles), at baseline and at the end of the trial. (a) Correlation between CSF AChE enzymatic activity levels and P‐tau(P‐181 form) at the beginning of the treatment. (b) Box plot represents the difference between CSF AChE enzymatic activity levels at baseline and at the end of the treatment expressed as % over basal levels. *Significantly different (*p* <.05) from the control group, as assessed by the Student's *t* test. (c) Correlation of the changes in CSF AChE activity (panel B) with the changes on P‐tau levels (Figure 4) considering all AD patients (solid line), only placebo‐treated (small dotted line) or only tideglusib (long dotted line)

AChE levels before tideglusib treatment were similar in the patients that received the placebo or tideglusib treatment (27.23 ± 6.46 and 22.60 ± 3.30, respectively). Patients with rivastigmine treatment displayed significantly lower AChE activity levels (11.46 ± 1.54) than those treated with galantamine (33.01 ± 2.79) or donepezil (30.03 ± 11.85). Interestingly, AChE activity levels correlated with P‐tau levels (*n* = 19 subjects; *r* = 0.525; *p* =.021: Figure 6a) and this correlation was higher when patients under rivastigmine treatment were excluded (*n* = 12 subjects; *r* = 0.709; *p* = .010). The baseline correlations of AChE activity with T‐tau (*r* = 0.44; *p* =.06), and with Aβ42 (*r* = 0.027; *p* =.91) were non‐significant. Interestingly, placebo‐treated patients had a significant increase in AChE activity after treatment (35 ± 16%; *p* =.04: Figure 6b), whereas those treated with tideglusib did not display significant changes from baseline levels (5 ± 3% increase; *p* = .94: Figure 6b). This steady AChE activity during the trial was independent of the prescribed AChE‐I (ANOVA test comparison of the three sub‐groups: *p* =.24). A positive correlation was found between the change in P‐tau levels and AChE activity prior and after treatment, across all the patients (*n* = 19; *r* = 0.768; *p* <.001). The correlation was also observed for the placebo‐treated patients (*n* = 5; *r* = 0.949; *p* =.014) although only showed a non‐significant tendency in the tideglusib‐treated subgroup alone (*n* = 14, *r* = 0.450; *p* =.10: Figure 6c). This tendency increased when the tideglusib‐treated patients under rivastigmine medication were excluded from the analysis (*n* = 8 subjects, *r* = 0.661; *p* =.074: data not shown).

## 4 DISCUSSION

This study demonstrated that an increased phosphorylation of tau by the kinase GSK3β can modulate the levels of the cholinergic AChE. These changes may consequently compromise cholinergic neurotransmission. Nonetheless, we cannot discard that GSK3β also exerts a cholinergic regulatory effect not related with P‐tau, a possibility that should be explored. Anyhow, our results are in accordance with a previous report where we showed that in a mouse model of FTDP17 tauopathy (Tg‐VLW), there are higher levels of AChE‐T protein, enzymatic activity and transcript levels than in wild‐type background strain mice (Silveyra, et al., 2012). However, other authors have reported preserved AChE levels in another mouse model of tauopathy, the THY‐Tau22 mice (García‐Gómez et al., 2016), suggesting that tau mutations may have different effects on AChE expression.

In this study, we induced an increase in phosphorylated tau in SH‐SY5Y cells by over‐expressing GSK3β together with wild‐type tau. Although increases in tau have been related to cell death and toxicity, tau over‐expression in our cell models seems not to be toxic since cell viability was not diminished. It is possible that the intracellular tau concentration might be regulated by its secretion to the extracellular medium via membrane vesicles (Simon et al., 2012), that do not induce significant cell death.

We demonstrate that the AChE activity and protein increases in cells in which wild‐type tau phosphorylation is increased, thereby mimicking what occurs in AD. Indeed, tau phosphorylation was inhibited by a specific GSK3β inhibitor, SB216763, which abolished the enhanced AChE activity and protein in SH‐SY5Y cells, and in mouse primary cell cultures. Likewise, over‐expression of a kinase‐dead mutant GSK3β that not incremented tau phosphorylation did not altered AChE expression. Over‐expression of the tau‐VLW mutation reinforces the role of P‐tau on AChE expression as the VLW mutation, as well as other tau mutations, make tau a more favorable substrate for brain protein kinases, favoring its hyperphosphorylation. This VLW mutation increases the hyperphosphorylation of tau at phosphothreonine 231 and phosphoserines 199/202 (Lim *et al*. 2001). We could not discard that tau phosphorylation by other kinases that are implicated in AD, such as CDK5 (Liu et al., 2016), AMPK (Tu et al., 2014), MAPKs (Zu et al. 2001), could also affect AChE expression, like GSK3β. However, further studies will be necessary to clarify this issue. The analysis of AChE levels in a small sample of AD patients undergoing a clinical trial with the GSK3β inhibitor tideglusib showed a similar but inconclusive tendency since only a minor not statically significant reduction was noticed for P‐tau levels. However, our data suggest that there may be cross‐talk between P‐tau and AChE.

In this study, AChE protein levels were analyzed by SDS‐PAGE/western blotting using an antibody that recognizes all AChE variants and with antibodies exclusive for specific AChE variants. The N‐19 antibody, which recognizes all AChE variants, revealed an immunoreactive pattern with a predominant band of 66 kDa and a faint band of 55 kDa. Differences in molecular mass of the AChE immunoreactive bands may reflect post‐translational processing, such as glycosylation, since native AChE splice variants are not predicted to have differential molecular mass (García‐Ayllón et al., 2010). We found that the 66 kDa species increases in cells with enhanced P‐tau levels as a result of GSK3Β and tau over‐expression. The identity of the 66 kDa species as the cholinergic AChE‐T variant was assessed using a specific antibody. No changes were observed in the immunoreactive levels of AChE‐R and N‐AChE variants, also assessed by specific antibodies. The specific increase in AChE‐T expression was corroborated by the analysis of transcripts levels by *q*RT‐PCR. Moreover, analysis in RA‐differentiated SH‐SY5Y cells also corroborated that the increase in AChE affects the true cholinergic species, a tetrameric form composed of AChE‐T subunits.

RA‐differentiated SH‐SY5Y cells can be considered as a model of cholinergic neurons since differentiation induces the expression of cholinergic markers (de Medeiros et al., 2019). In this cellular model, we also assessed whether the increase in AChE mediated by tau phosphorylation could lead to a cholinergic imbalance since an alteration in cholinergic AChE levels with no corresponding increase in ChAT could potentially lower levels of the neurotransmitter ACh. Decreased ACh levels have been previously described in rat striatum as result of GSKβ activation (Zhao et al., 2013). This activation leads to an inhibition of ChAT activity as result of an altered cellular distribution. Moreover, the number of ChAT‐positive neurons is also decreased in the medial septum of old Tg601 mice, a model of tauopathy (Hara et al., 2017), whereas the unchanged levels of ChAT in transgenic models of mutated tau have been reported in other studies (García‐Gómez et al., 2016; Silveyra, et al., 2012).

The alteration in cellular ACh levels may have physiopathological consequences. In AD, decreased ACh is linked to impaired cognition, behavior, and daily living activities. A previous report from our group using a rat model of liver cirrhosis showed that impairment of the brain cholinergic system as a result of increased AChE and reduced ACh may be associated with failure in learning and memory functions (Garcia‐Ayllon et al., 2008).

As previously stated, the AChE‐T variant is the main cholinergic species, but it can develop other roles not related with synaptic neurotransmission. Studies have also shown that GSK3β can regulate the expression of AChE‐T, for example, during apoptosis caused by calcium dyshomeostasis (Jing et al., 2007). The regulation of AChE‐T during apoptosis (Zhang et al., 2002) may be driven by specific mechanisms. Indeed, it was shown that GSK3β may stabilize AChE‐T protein preventing its proteasomal degradation in HEK cells (Jing et al., 2013). GSK3β revealed multifaceted roles also related with transcriptional regulation (Lauretti et al., 2020). Thus, we cannot discard that GSK3β also modulates AChE expression by other mechanisms independent of changes in tau phosphorylation.

The increase in AChE‐T may also be a consequence of the increase in intracellular calcium. AChE‐T expression is modulated by AChE promoter activity as a result of perturbations in intracellular calcium homeostasis (Luo et al., 1994; Zhu et al., 2007) and extracellular tau can induce an increase in intracellular calcium in cultured neuronal cells, probably via M1 and M3 muscarinic receptors (Gómez‐Ramos et al., 2008). Moreover, an increment of intracellular calcium could also trigger an enhanced AChE promoter activity as a result of the binding of CCAAT‐binding factor (CBF/NF‐Y) to the CCAAT motif within AChE promoter. Thus, the increase in AChE‐T could also be related to the alterations of the binding to transcription‐binding sites within the AChE promoter region such as Sp‐1, cyclic AMP‐responsive element, AP‐1, and NFAT that could be regulated by tau phosphorylation and GSK3β. Indeed, the deposition of phosphorylated tau has been related to an increased c‐Jun, c‐Fos, and CREB‐1 expression in neurons in Picks disease brains (Nieto‐Bodelón et al., 2006). In this regard, binding of c‐jun to the AP1 site of AChE promoter has been related to an increased AChE‐T in PC12 cells (Zhang et al 2008).

AChE‐T over‐expression has been linked to an increase in neurodegeneration (Farchi et al., 2007) and programmed cell death (Greenberg et al., 2010; Toiber et al., 2009). Interestingly, it has been reported that AChE‐T facilitates Aβ fibril formation and AD plaque formation (Berson et al., 2008). Indeed, transgenic mice over‐expressing AChE‐T and the APP Swedish mutation, show early deposition and more abundant β‐amyloid plaques than mice over‐expressing mutant APP Swedish mutation alone (Rees et al., 2005). We speculate that AChE‐T is part of a vicious circle in which Aβ deposition may be potentiated as a result of an increase in AChE‐T by P‐tau.

Several GSK3β inhibitors are currently being tested for the treatment of AD. Tideglusib is an irreversible GSK3β inhibitor, which reduces tau phosphorylation and prevents apoptotic death in human neuroblastoma cells and murine primary neurons (Domínguez et al., 2012). In AD patients treated with tideglusib a trend toward a cognitive benefit of 1.68 points on the MMSE screen and ADAS‐Cog test battery was reported in a pilot study (del Ser et al., 2012). In a subsequent 26‐week Phase II clinical trial (ARGO study), tideglusib did not show clinical efficacy (Lovestone et al., 2015), although some clinical trends in patients treated with low doses of tideglusib have been demonstrated. Our study was conducted using CSF samples from a small group of AD patients enrolled in the second phase II clinical trial. In our sample collection, according to results of the ARGO study (Lovestone et al., 2015), tideglusib‐treated patients showed a trend toward decreased CSF P‐tau levels as compared with placebo‐treated patients that was not statistically significant. This result might suggest that tideglusib does not act as a GSK3β inhibitor, yet the authors indicated that an inhibitory effect was present since there was a significant decrease in CSF BACE1 relative to the patients that received the placebo. This alteration to BACE1 is in accordance with the reduction observed in an AD transgenic model treated with lithium, suggesting an inhibitory effect of the drug. There were no changes in any of the other core AD biomarkers T‐tau and Aβ1‐42. Importantly, patients enrolled in this clinical trial were under AChE‐I treatment for at least 4 months prior to baseline and maintained this AChE‐I treatment during the trial. AChE activity in CSF is differently affected by AChE‐Is; rivastigmine causes persistent inhibition of AChE (Darreh‐Shori et al., 2002; Nordberg et al., 2009; Parnetti et al., 2011), whereas donepezil and galantamine cause a rebound increase in CSF AChE activity (García‐Ayllón et al., 2007; Nordberg et al., 2009; Parnetti et al., 2011). In agreement with these data, AChE activity levels at baseline were lower in our rivastigmine‐treated patients than in those under donepezil or galantamine treatment. The influence of AChE‐I on CSF AChE activity may affect the interpretation of a potential effect of tideglusib on AChE levels. While AChE levels at baseline correlate with P‐tau, suggesting that P‐tau could influence this enzyme, a cause‐effect relationship has not been demonstrated. A positive correlation was also observed for the placebo‐treated patients between the change in P‐tau levels and AChE activity during the trial. Interestingly, the levels of CSF AChE were still increased in the placebo group during the 26 weeks of trial (for four out of the five patients, two receiving donepezil, one galantamine and another one rivastigmine; one patient under donepezil treatment did not change). Tideglusib‐treated patients displayed similar CSF AChE activity levels at the end of the trial, compared with baseline. This result suggests that even if the GSK3β inhibition by tideglusib fails to drive significant changes in CSF P‐tau, it is able to act on CSF AChE activity. In a neurodegenerative mouse model over‐expressing transgenic tau, the suppression of tau expression resulted in improved memory function although NFTs accumulation persisted (Santacruz et al., 2005). Thus, the pathological and therapeutic effect of changes in tau hyperphosphorylation should be evaluated not only in terms of tau pathology. More studies are needed to clarify the potential effect of tideglusib on AChE activity and cholinergic function, if possible, in early diagnosed AD patients without previous treatment with AChE‐I.

Taken together our findings point to a possible influence of tau hyperphosphorylation on cholinergic AChE activity that could be relevant in the physiopathology of AD. Therefore, the early increase in AChE expression that occurs around NFT (Mesulam et al., 1987) may be a consequence of disturbed tau phosphorylation. Indeed, we have shown in cellular models the colocalization of P‐tau and AChE in cytoplasmatic regions like in neurons of the Tg‐VLW mutant (Silveyra, et al., 2012). Moreover, it has been reported that AChE activity may be preserved or even increased in some brain areas of patients with mild AD (Herholz et al., 2004). Additionally, the study supports the view that early tau hyperphosphorylation may cause an impairment of cholinergic activity with a decrease in ACh levels that may be a contributing factor for the degeneration of cholinergic neurons of the basal forebrain. Finally, our results indicated the relevance of measuring CSF AChE as a biomarker for trials with GSK3β inhibitors, and the need to analyze the cholinergic enzymes and neurotransmitter in future studies using animal models or clinical trials.

## CONFLICT OF INTEREST

None of the authors have any actual or potential financial conflicts or conflicts of interest related with this study or with any AChE‐I drug or GSK‐3 inhibitor.

### Open Research Badges

This article has received a badge for *Open Materials* because it provided all relevant information to reproduce the study in the manuscript. More information about the Open Practices badges can be found at https://cos.io/our‐services/open‐science‐badges/.

## Supporting information

Fig S1‐S3

Supplementary Material
