# Supplementary material for: Tau phosphorylation by glycogen synthase kinase 3β modulates enzyme acetylcholinesterase expression
Source: J Neurochem. 2020 Oct 4;157(6):2091–105. doi: 10.1111/jnc.15189 (PMC8359467; doi:10.1111/jnc.15189)
Supplement: Supplementary file 1 — Fig S1‐S3 [file JNC-157-2091-s001.pdf]

Tau phosphorylation by glycogen synthase kinase 3β modulates AChE expression

María-Ángeles Cortés-Gómez[1,2,3], Esther Llorens-Álvarez[1,3], Jordi Alom[2,4], Teodoro del Ser[5], Jesús Avila[2,6], Javier Sáez-Valero[2,3] and María-Salud García-Ayllón[1,2,3]*.

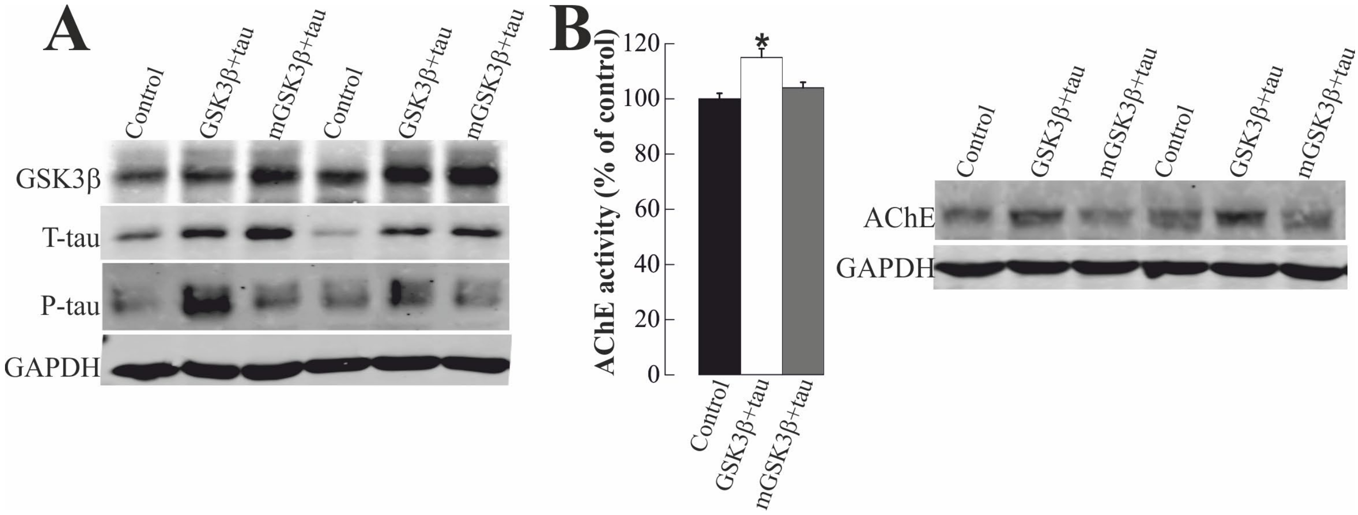

Supplemental Figure 1. Overexpression of a kinase dead GSK3β mutant does not alter phosphorylated tau levels and AChE expression. SH-SY5Y cells were transfected with DNA vectors that encode wild type GSK3β and tau (GSK3β+tau) or with a kinase dead GSK3β mutant and wild type tau (mGSK3β+tau) or with a pCI empty control vector (Control). **(A)** Western blot of proteins from cellular extracts were probed with antibodies against GSK3β, tau, phosphorylated tau (P-tau). GAPDH was used as housekeeping. Representative blots are shown. Overexpression of the mGSK3β+tau does not alter P-tau levels respect to PCI control. **(B)** AChE enzymatic activity (% relative to PCI control) and protein (representative blot is shown) were also determined in cellular extracts. Only in cellular extracts from GSK3β+tau transfected cells AChE levels were increased respect to controls. Results were confirmed in n = 18 independent cell determinations (obtained from 3 independent cell sets of experiments). Represented values are means ± SEM. *Significantly different ($p < 0.05$) from control group, as assessed by one-way ANOVA followed by Tukey test for pair-wise comparisons.

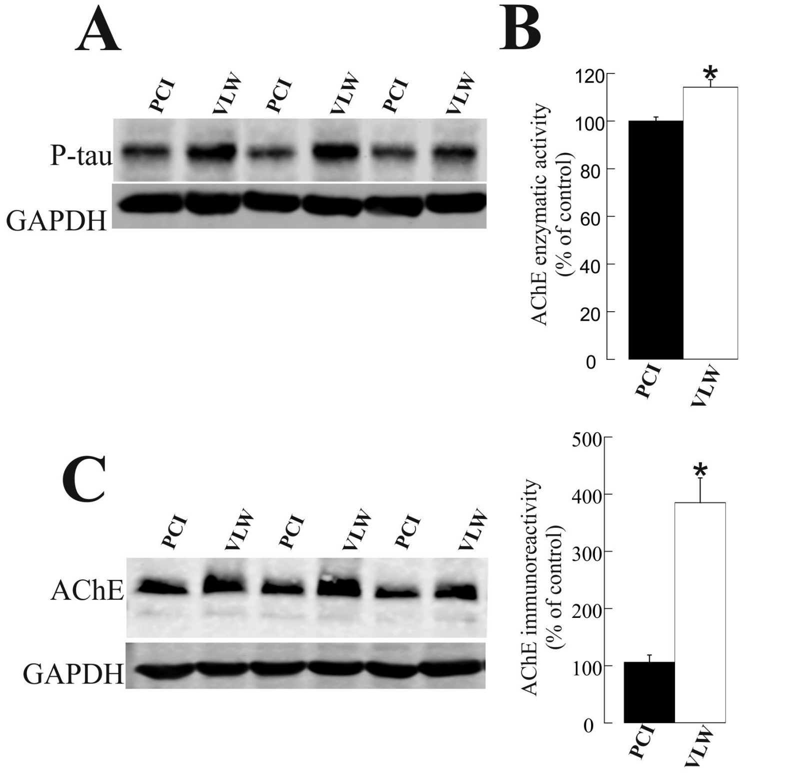

Supplemental Figure 2. Over-expression of VLW mutant tau increases AChE expression in SH-sy5y cells. (A) Over-expression of VLW mutant tau resulted in increased phosphorylated tau (P-tau) levels (blotted with AT8 antibody) as compared with control cells transfected with a PCI "empty" vector. A representative blot is showed. **(B)** Increased AChE enzymatic activity were detected in cells that overexpress the VLW mutation. Data represent percentage (%) respect to control. **(C)** AChE protein is increased in VLW transfected cells. Representative blot of ACHE probed with N-19 antibody an densitometric quantification of AChE immunoreactive bands showed the increment relative to PCI control cells. Data represent percentage (%) respect to control. Mean value ± SEM are represented, from at least of 18 independent determinations from three independent experiments. *Significantly different ($p < 0.05$) from the control group, as assessed by the Student's *t* test.

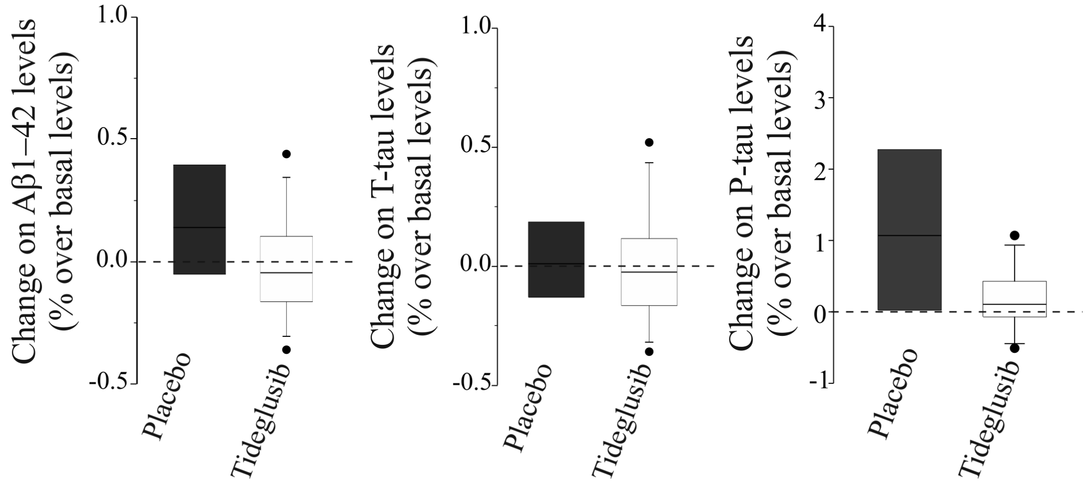

Supplemental Figure 3. Difference in CSF levels of core AD biomarkers prior to and after 26 weeks of clinical trial with tideglusib. CSF from placebo (n= 5) and tideglusib (n=14; 1000 mg/day see regiment of administration in Table 1) treated AD patients were obtained at the baseline and end of the clinical trial, after 26 weeks. Levels of the core AD biomarkers, β-amyloid 1-42 (Aβ1-42), total tau (T-tau) and phosphorylated tau (P-tau, P181 form) were measured in CSF samples and differences between levels before and after treatment were calculated. Box plot of the change expressed as percentage (%) over basal levels were represented. None of the differences were significant as assessed by the Student's *t* test.
